# Protocol for generating and selecting transgenic tomato lines through *Agrobacterium tumefaciens*-mediated transformation

**DOI:** 10.1016/j.xpro.2025.104322

**Published:** 2026-01-10

**Authors:** Fanourios Mountourakis, Sotirios Fragkostefanakis, Panagiotis Nikolaou Moschou

**Affiliations:** 1 Department of Biology, University of Crete, 70013 Heraklion, Greece; 2 Institute of Molecular Biology and Biotechnology, Foundation for Research and Technology-Hellas, 70013 Heraklion, Greece; 3 Institute of Biosciences, Molecular Cell Biology of Plants, Goethe University, Frankfurt am Main, Germany; 4 Department of Molecular Sciences, Uppsala BioCenter, Swedish University of Agricultural Sciences and Linnean Center for Plant Biology, Uppsala, Sweden

**Keywords:** plant sciences, tissue engineering, biotechnology and bioengineering

## Abstract

Here, we present a protocol for generating and selecting stable transgenic tomato lines through *Agrobacterium tumefaciens*-mediated transformation of cotyledon and hypocotyl explants. We describe steps for sterilizing and planting tomato seeds, cotyledon and hypocotyl excision and preculture, *Agrobacterium tumefaciens* preparation and co-cultivation with explants, and recovering explants and bacterial overgrowth restriction. We then detail procedures for selecting green calli; regenerating explant excision and growth in non-selective media; plant hardening, potting, and growing; and T-DNA insertion confirmation by PCR.

For complete details on the use and execution of this protocol, please refer to Mountourakis et al.^1^^,^^2^^,^^3^

## Highlights

• Steps for generating stable transgenic tomato lines in less than 6 months
• Instructions for tomato explant regeneration using 1 hormone
• Guidance on *Agrobacterium* elimination using Timentin
• Procedures for T-DNA insertion confirmation by PCR and foliar spray

## Abstract (editor highlights)

Publisher’s note: Undertaking any experimental protocol requires adherence to local institutional guidelines for laboratory safety and ethics.

## Graphical abstract

**Figure:**
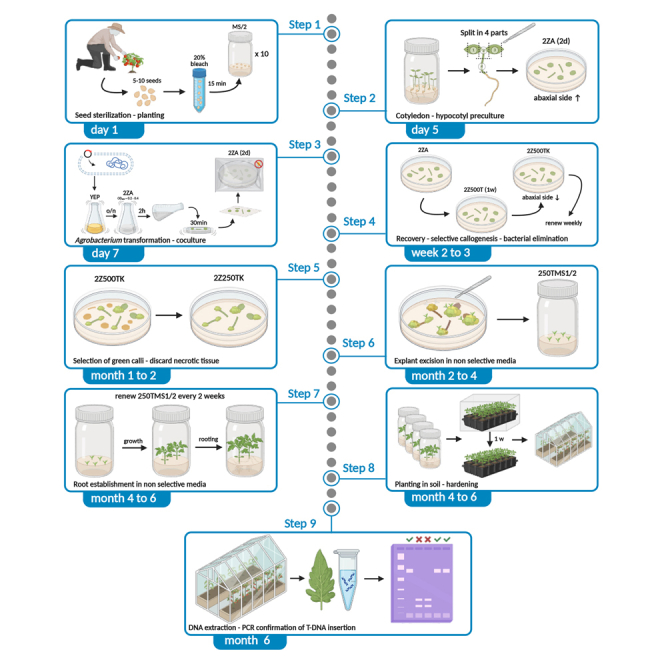

## Before you begin

Tissue culture remains a fundamental and indispensable technique in contemporary plant biotechnology due to its proven capacity to provide reproducible regeneration of genetically uniform and stable transgenic plants. Despite advances in genome editing methods like virus-induced techniques that can reduce reliance on tissue culture, it is still the gold standard for crops like tomato. The protocol below describes the specific steps for *Agrobacterium tumefaciens*-mediated stable transformation of tomato, optimized for the cultivar MicroTom and Kanamycin selection marker. However, we have efficiently applied the same protocol for Hygromycin B selection marker and other tomato cultivars like Moneymaker, obtaining similar results in terms of time efficiency and successful generation of transgenic lines.

### Innovation

Since the first reported application of *Agrobacterium* *tumefaciens*-mediated transformation of *Solanum lycopersicum* over thirty years ago,^1^ numerous improvements have been proposed and published. These advancements, including optimization of explant types, bacterial strains, hormone use, and selection protocols, have enabled successful transformation across different cultivars/genotypes, thus facilitating genome editing applications in tomato.^2^ However, due to the time-consuming and labor-intensive nature of many different, overly complicated tissue culture protocols, researchers are hesitant to convert their basic research from *Arabidopsis thaliana* to this economically important species. In this study, we have refined conventional protocols aiming at rapid, easy, and cost-efficient generation of transgenic lines. Unlike common protocols that recommend the use of multiple hormones such as various auxins and gibberellins,^4^^,^^5^ we used only *trans*-zeatin to induce callogenesis and plantlet regeneration. In addition, during explant establishment and rooting stages, no hormones or selection antibiotics were applied, which significantly increased survival and successful rooting, without any increase in the frequency of spurious regenerants. This approach, reported here for the first time to our knowledge, saved several weeks without increasing the number of “escape plants”. We employed a strategy of gradual Timentin™ reduction to effectively suppress Agrobacterium overgrowth, even in the naturally ampicillin-insensitive GV3101 strain. Furthermore, tomato plants naturally have elevated levels of auxins that facilitate self-induced rooting.^3^ We enhanced this response with Timentin™, whose degradation products include an auxin analog recently demonstrated to promote organogenesis.^6^ In essence, this protocol requires researchers to acquire only two specific reagents [*trans-*zeatin and Timentin™], while the remaining materials are commonly available.

### Institutional permissions

The “European Research Council Executive Agency Ethics Sector Committee” under the call ERC-2023-COG (Grant 101126019 – PLANTEX) and the institutional “Ethics and Deontology Committee of Foundation for Research and Technology - Hellas” approve the experiments done here and confirm that they conform to the relevant regulatory standards of European Union and Greece. Ensure you obtain and maintain thorough institutional biosafety approval prior to commencing any work involving *Agrobacterium tumefaciens* and the generation of transgenic plants, adhering strictly to all relevant guidelines, regulations, and containment procedures to guarantee safe and responsible experimental practices.

### Preparation of stock solutions

**Timing: 1 h**

1. Prepare the following stock solutions (1,000x concentrated):
  a. *Trans*-zeatin: 2 mg/mL in water (filter sterilize, store at −20°C).
  b. Acetosyringone: 100 mM in dimethyl sulfoxide - DMSO (store at −20°C).
  c. Timentin™: 500 mg/mL in water (filter-sterilize, store at −20°C).
  d. Commonly used selection antibiotics: Rifampicin in DMSO 25 mg/mL (light sensitive, store at −20°C), Gentamycin 50 mg/mL in water (filter-sterilize, store at −20°C) Kanamycin: 50 mg/mL in water (filter-sterilize, store at −20°C), Hygromycin B: 5 mg/mL (light sensitive, filter-sterilize, store at −20°C).

***Note:*** Prepare all stock solutions under sterile conditions. Filter-sterilize heat-labile components using 0.2 μm PES filters. Protect light sensitive components using aluminum foil or black tubes.

### Preparation of germination medium: Murashige and Skoog—MS/2 medium

**Timing: 1 h**

2. Prepare the liquid MS/2 media.
  a. Weigh 2.15 g of MS salts powder stock and add to a 1 L glass beaker.
  b. Add 900 mL of Milli-Q water and mix thoroughly using a magnetic stirrer.
  c. Adjust pH to 5.8 using 1 M KOH.
  d. Fill up to 1 L with Milli-Q water.
  e. Distribute evenly in 10 200 mL glass bottles (100 mL in each bottle).
  f. Sterilize the solution using an autoclave (liquid cycle, 121°C for 20 min) and allow to cool down below 50°C before proceeding.

***Note:*** For solid MS/2, weight and add 0.6 g plant agar per 100 mL before step f. Sterile solid and liquid MS/2 media can be stored at 23±2°C for several weeks. A carbon source such as sucrose is not advised in tomato seedling germination.

### Preparation of Yeast Extract Peptone—YEP medium

**Timing: 1 h**

3. Prepare the liquid YEP (Yeast Extract Peptone) media.
  a. Weigh 10 g of yeast extract powder and add to a 1 L glass bottle.
  b. Add 900 mL of Milli-Q water and mix thoroughly using a magnetic stirrer.
  c. Weigh and add 10 g of peptone powder.
  d. Weigh and add 5 g of NaCl.
  e. Adjust pH to 7 using 1M KOH.
  f. Fill up to 1 L with Milli-Q water.
  g. Distribute evenly in ten 200 mL glass bottles (100 mL in each bottle).
  h. Sterilize the solution using an autoclave (liquid cycle, 121°C for 20 min) and allow to cool down below 50°C before proceeding.
  i. Add the appropriate antibiotics to the medium as required.

***Note:*** In our case we used Rifampicin 25 μg/mL (resistance gene at the C58 chromosomal backbone of GV3101), Gentamycin 50 μg/mL (resistance gene at the pMP90 helper plasmid /pTiC58DT-DNA), Kanamycin 50 μg/mL (resistance gene at the pICH86988 T-DNA plasmid). For solid YEP media, weight and add 1.5 g agar before per 100 mL step h. Sterile solid and liquid YEP media can be stored at 23±2°C for several weeks.

### Preparation of preculture—Co-cultivation medium (2ZA)

**Timing: 1 h**

4. Prepare the liquid MS/2 media.
  a. Weight and add 3 g of sucrose per 100 mL of liquid MS/2 media and mix well on a magnetic stirrer.
  b. Adjust pH to 5.5 using 1M KOH.
  c. Sterilize the solution using an autoclave (liquid cycle, 121°C for 20 min) and allow to cool down below 50°C before proceeding.
  d. Add 100 μL per 100 mL of *trans*-zeatin stock solution (final concentration 2 μg/mL).
  e. Add 100 μL per 100 mL of acetosyringone stock solution (final concentration 100 μM).

***Note:*** For solid 2ZA media, weight and add 0.6 g plant agar per 100 mL before step c. 2ZA should be prepared fresh. The solid 2ZA media be stored at 2°C–8°C for 1–2 weeks in the Petri dishes.

### Preparation of regeneration medium (2Z500T, 2Z500TK, and 2Z250TK)

**Timing: 1 h**

5. Prepare the liquid MS/2 media.
  a. Weight and add 3 g of sucrose per 100 mL of liquid MS/2 media and mix well on a magnetic stirrer.
  b. Sterilize the solution using an autoclave and allow to cool down below 50°C before proceeding.
  c. Add 100 μL per 100 mL of *trans*-zeatin stock solution (final concentration 2 μg/mL).
  d. Add antibiotics accordingly:
    i. For 2Z500T, add 100 μL per 100 mL of Timentin™ stock solution (final concentration 500 μg/mL).
    ii. For 2Z500TK, add 100 μL per 100 mL of Timentin™ stock solution (final concentration 500 μg/mL and add 100 μL per 100 mL of Kanamycin stock solution (final concentration 50 μg/mL).
    iii. For 2Z250TK, add 50 μL per 100 mL of Timentin™ stock solution (final concentration 250 μg/mL and add 100 μL per 100 mL of Kanamycin stock solution (final concentration 50 μg/mL).

***Note:*** For solid 2Z500T, 2Z500TK and 2Z250TK media, weight and add 0.6 g plant agar per 100 mL before step b. 2Z500T, 2Z500TK and 2Z250TK should be prepared fresh. The solid media can be stored at 2°C–8°C for 1–2 weeks in the petri dishes.

**CRITICAL:** We optimized this protocol for T-DNA inserts containing neomycin phosphotransferase II gene (*NPTII*, Kanamycin resistance gene) under the control of the common nopaline synthase (NOS) promoter. For T-DNA inserts containing aminoglycoside phosphotransferase (*APH(4)-Ia*, Hygromycin B resistance gene) under the control of the common Cauliflower Mosaic Virus (CamV) 35S promoter, 5 μg/mL Hygromycin B final concentration is enough for efficient selection.

### Preparation of rooting medium (250TMS1/2)

**Timing: 1 h**

6. Prepare the liquid MS/2 media.
  a. Weight and add 1 g of sucrose per 100 mL of liquid MS/2 media and mix well on a magnetic stirrer.
  b. Sterilize the solution using an autoclave and allow to cool down below 50°C before proceeding.
  c. Add 50 μL per 100 mL of Timentin™ stock solution (final concentration 250 μg/mL).:

***Note:*** For solid 250TMS1/2, weight and add 0.6 g plant agar per 100 mL before step b. 250TMS1/2 should be prepared fresh. The solid media can be stored at 2°C–8°C for 1–2 weeks in the glass jars.

## Key resources table

**Table:** 

REAGENT or RESOURCE	SOURCE	IDENTIFIER
**Bacterial and virus strains**		

*Agrobacterium tumefaciens* GV3101	GoldBio	Cat#CC-207

**Chemicals, peptides, and recombinant proteins**		

Acetosyringone	Apollo Scientific	Cat#BIA2203; CAS:2478-38-8
Agar	Sigma-Aldrich	Cat#05038; CAS:9002-18-0
Gentamycin sulfate	Sigma-Aldrich	Cat#345814-M; CAS:1405-41-0
Kanamycin monosulfate	Sigma-Aldrich	Cat#BP861; CAS:25389-94-0
Murashige & Skoog medium including vitamins	Duchefa Biochemie	Cat#M0222; CAS:N/A
Peptone - Bacto Peptone	Sigma-Aldrich	Cat#211677; CAS:91079-46-8
Plant agar	Duchefa Biochemie	Cat# P1001; CAS:9002-18-0
Rifampicin	Sigma-Aldrich	Cat#R3501; CAS:13292-46-1
Sodium chloride	Sigma-Aldrich	Cat#S9888; CAS:7647-14-5
Sucrose	Sigma-Aldrich	Cat#S0389; CAS:57-50-1
Timentin^TM^ Ticarcillin/Clavulanate (15/1)	GoldBio	Cat#T-104-100; CAS:4697-14-7; CAS:61177-45-5
*trans-*zeatin	GoldBio	Cat#Z-105; CAS:1637-39-4
Yeast extract	Sigma-Aldrich	Cat#Y1625; CAS:8013-01-2

**Experimental models: Organisms/strains**		

Tomato cultivar MicroTom	TOMATOMA	https://tomatoma.nbrp.jp Cat#TOMJPF00001
Tomato cultivar Moneymaker	PepperSeeds	https://pepperseeds.eu Cat# 612

**Other**		

CultureJar G9, Glass Plant Tissue Culture Vessel (220 mL)	Phytotech Labs	Cat#C1770
Closure, PhytoCap, CultureJar Closure	Phytotech Labs	Cat#C070
FITOCLIMA 1200 Plant Growth Chamber	Aralab	https://aralab.pt

## Materials and equipment

**Table:** Germination medium -Murashige and Skoog – MS/2 medium (pH=5.8, solid)

Reagent	Final concentration	Amount
Milli-Q water	N/A	1 L
MS salts	N/A	2.15 g
Plant agar	0.6% (w/v)	6 g
**Total**	**N/A**	**1 L**

Store at 23±2°C several weeks.

***Alternatives:*** If MS salts are not readily available, the complete recipe is as follows:

**Table:** 

Reagent	Final concentration	Amount
Milli-Q water	N/A	1 L
CaCl_2_	2.99 mM	0.332 g
KH_2_PO_4_	1.25 mM	0.17 g
KNO_3_	18.79 mM	1.90 g
MgSO_4_	1.50 mM	0.181 g
NH_4_NO_3_	20.61 mM	1.65 g
CoCl_2_.6H_2_O	0.11 μM	0.025 mg
CuSO_4_.5H_2_O	0.10 μM	0.025 mg
FeNaEDTA	100.00 μM	36.70 mg
H_3_BO_3_	100.27 μM	6.20 mg
KI	5.00 μM	0.83 mg
MnSO_4_.H_2_O	100.00 μM	16.90 mg
Na2MoO_4_.2H_2_O	1.03 μM	0.25 mg
ZnSO_4_.7H_2_O	29.91 μM	8.60 mg
Glycine	26.64 μM	2.00 mg
Myo-Inositol	554.94 μM	0.10 g
Nicotinic acid	4.06 μM	0.50 mg
Pyridoxine - HCl	2.43 μM	0.50 mg
Thiamine - HCl	0.30 μM	0.10 mg
Plant agar	0.6%	6 g
**Total**	**N/A**	**1 L**

**Table:** Yeast Extract Peptone medium (pH=7, liquid)

Reagent	Final concentration	Amount
Milli-Q water	N/A	1 L
Yeast extract	1% (w/v)	10 g
Peptone	1% (w/v)	10 g
NaCl	0.5% (85.6 mM)	5 g
**Total**	**N/A**	**1 L**

Store at 23±2°C several weeks. Upon addition of antibiotics store at 4°C for up to 2 weeks (protected from light).

**CRITICAL:** Add the appropriate antibiotics to the medium as required. In our case we used Rifampicin 25 μg/mL (resistance gene at the C58 chromosomal backbone of GV3101), Gentamycin 50 μg/mL (resistance gene at the pMP90 helper plasmid /pTiC58DT-DNA), Kanamycin 50 μg/mL (resistance gene at the pICH86988 T-DNA plasmid).

**Table:** Yeast Extract Peptone medium (pH=7, solid)

Reagent	Final concentration	Amount
Milli-Q water	N/A	1 L
Yeast extract	1% (w/v)	10 g
Peptone	1% (w/v)	10 g
NaCl	0.5% (85.6 mM)	5 g
Agar	1.5% (w/v)	1.5 g
**Total**	**N/A**	**1 L**

Store at 23±2°C several weeks. Upon addition of antibiotics store at 4°C for up to 2 weeks (protected from light).

**CRITICAL:** Add the appropriate antibiotics to the medium as required. In our case we used Rifampicin 25 μg/mL (resistance gene at the C58 chromosomal backbone of GV3101), Gentamycin 50 μg/mL (resistance gene at the pMP90 helper plasmid /pTiC58DT-DNA), Kanamycin 50 μg/mL (resistance gene at the pICH86988 T-DNA plasmid).

**Table:** Preculture – Co-cultivation medium (2ZA) (pH = 5.5, liquid)

Reagent	Final concentration	Amount
Milli-Q water	N/A	100 mL
MS salts	N/A	0.22 g
Sucrose	3% (87.7 mM)	3 g
*trans*-zeatin (conc. 2 mg/mL)	2 μg/mL (9.12 μΜ)	100 μL
Acetosyringone (conc. 100 mM)	100 μΜ	100 μL
**Total**	**N/A**	**100 mL**

Store at 4°C in the petri dishes for up to 2 weeks.

**Table:** Preculture – Co-cultivation medium (2ZA) (pH = 5.5, solid)

Reagent	Final concentration	Amount
Milli-Q water	N/A	100 mL
MS salts	N/A	0.22 g
Sucrose	3% (87.7 mM)	3 g
*trans*-zeatin (conc. 2 mg/mL)	2 μg/mL (9.12 μΜ)	100 μL
Acetosyringone (conc. 100 mM)	100 μΜ	100 μL
Plant agar	0.6%	0.6 g
**Total**	**N/A**	**100 mL**

Store at 4°C in the petri dishes for up to 2 weeks.

**Table:** Regeneration medium (2Z500T) (pH = 5.8, solid)

Reagent	Final concentration	Amount
Milli-Q water	N/A	100 mL
MS salts	N/A	0.22 g
Sucrose	3% (87.7 mM)	3 g
*trans*-zeatin (conc. 2 mg/mL)	2 μg/mL (9.12 μΜ)	100 μL
Timentin™ (conc. 500 mg/mL)	500 μg/mL	100 μL
Plant agar	0.6%	0.6 g
**Total**	**N/A**	**100 mL**

Store at 4°C in the petri dishes for up to 2 weeks.

**Table:** Regeneration medium (2Z500TK) (pH = 5.8, solid)

Reagent	Final concentration	Amount
Milli-Q water	N/A	100 mL
MS salts	N/A	0.22 g
Sucrose	3% (87.7 mM)	3 g
*trans*-zeatin (conc. 2 mg/mL)	2 μg/mL (9.12 μΜ)	100 μL
Timentin (conc. 500 mg/mL)	500 μg/mL	100 μL
Kanamycin (conc. 50 mg/mL)	50 μg/mL (85.82 μΜ)	100 μL
Plant agar	0.6% (w/v)	0.6 g
**Total**	**N/A**	**100 mL**

Store at 4°C in the petri dishes for up to 2 weeks.

**CRITICAL:** Add the appropriate antibiotics to the medium as required. In our case we used Kanamycin 50 μg/mL (resistance gene at the pICH86988 T-DNA plasmid). If you get a lot of untransformed “escape plants”, increase Kanamycin to 100 μg/mL. We have also used Hygromycin B 5 μg/mL successfully although we highly recommend Kanamycin over Hygromycin B when working with tomato. We suggest the same x2 increase with the cultivar Moneymaker.

**Table:** Regeneration medium (2Z250TK) (pH = 5.8, solid)

Reagent	Final concentration	Amount
Milli-Q water	N/A	100 mL
MS salts	N/A	0.22 g
Sucrose	3% (87.7 mM)	3 g
*trans*-zeatin (conc. 2 mg/mL)	2 μg/mL (9.12 μΜ)	100 μL
Timentin™ (conc. 500 mg/mL)	250 μg/mL	50 μL
Kanamycin (conc. 50 mg/mL)	50 μg/mL (85.82 μΜ)	100 μL
Plant agar	0.6% (w/v)	0.6 g
**Total**	**N/A**	**100 mL**

Store at 4°C in the petri dishes for up to 2 weeks.

**CRITICAL:** Add the appropriate antibiotics to the medium as required. In our case, we used Kanamycin 50 μg/mL (pICH86988 T-DNA binary plasmid). If many untransformed escape plants appear, increase Kanamycin to 75–100 μg/mL. We have also used Hygromycin B at 5 μg/mL successfully; however, we highly recommend Kanamycin over Hygromycin B when working with tomato. For the cultivar Moneymaker, we recommend the same 2-fold increase in Kanamycin concentration (to 100 μg/mL).

**Table:** Rooting medium (250TMS1/2)

Reagent	Final concentration	Amount
Milli-Q water	N/A	100 mL
MS salts	N/A	0.22 g
Sucrose	1% (29.22 mM)	1 g
Timentin™ (conc. 500 mg/mL)	250 μg/mL	50 μL
Plant agar	0.6% (w/v)	0.6 g
**Total**	**N/A**	**100 mL**

Store at 4°C in the petri dishes for up to 2 weeks.

## Step-by-step method details

### Sterilization and planting of tomato seeds

**Timing: 5–7 days**

In this step, we describe how to initiate the protocol by collecting, sterilizing and planting tomato seeds.

***Note:*** In this protocol, you should work under a laminar flow hood to prevent contamination of your samples. All materials and surfaces should be properly disinfected with 70% (v/v) ethanol solution before beginning any procedure, and only sterilized tools and equipment should be used. Stainless steel equipment such as scalpels can be sterilized immediately before use using a Bunsen burner, while single-use equipment such as pipette tips should be autoclaved prior to use. Hands should be washed thoroughly, and gloves, lab coats, and other protective gear must always be worn. Movements should be deliberate and minimal to avoid disrupting airflow or introducing contaminants. Maintaining focus and discipline throughout the process ensures the integrity of the work and prevents contamination of samples, equipment, and the surrounding environment.

1. Collect the tomato seeds from a red ripe tomato or start by using freshly dried seeds (less than 3–4 years old).

***Note:*** Tomato seeds often exhibit primary physiological dormancy that can persist for several weeks. This can reduce germination rates and/or delay germination.

2. Prepare a 50 mL Falcon centrifuge tube with 25 mL of 20% (v/v) diluted commercial bleach (containing 4.5% NaOCl) and 0.05% (v/v) Tween20.
3. Add approximately 100 tomato seeds to the solution and mix thoroughly for 15 min using an orbital shaker.
4. Remove the bleach solution by decanting and wash seeds once with 25 mL of 70% (v/v) ethanol for 1 min.
5. Remove the 70% (v/v) ethanol solution by decanting and wash at least 3 times with sterile dH_2_0 for 1 min.
6. Fill 10–20 sterile Glass Plant Tissue Culture Vessel like CultureJar G9 or similar with 10-20 mL solid Germination Medium – MS/2.

***Note:*** 12 cm × 12 cm square Petri dishes can be used in this step but tomato growth in jars sparsely planted is more robust as the plants lack signs of stress.

7. Distribute 5–10 seeds per jar.
8. Let the tomato plants grow in controlled environment plant growth chamber at 23°C–25°C, with a 16/8 light/dark photoperiod, and approximately 100 μmol/m^2^ s full spectrum light for 5-7 days.

**CRITICAL:** The plants should reach the point where the cotyledons are fully developed, and the first true leaves are just visible. When the first true leaves are fully developed, transformation efficiency drops significantly.^7^ This time point varies between among cultivars and growth conditions, so it is advisable that you optimize it at your own setup before starting your experiments.

### Cotyledon and hypocotyl excision and preculture

**Timing: 2 days**

In this step, we describe how to correctly excise the cotyledons and hypocotyls from the tomato seedlings while preparing the tissue for bacterial infection. Preculture – Co-cultivation medium (2ZA) is used because sugars, low pH, and acetosyringone have been reported to increase the efficiency of *Agrobacterium tumefaciens*-mediated plant transformation.^8^ Here, we add *trans*-zeatin, a type of naturally occurring cytokinin that stimulates cell division, shoot formation, and, in tomato, *de novo* callogenesis.^4^

9. Fill up common Petri dishes (92 mm diameter x 16 mm height or similar) with freshly prepared solid Preculture – Co-cultivation medium (2ZA).
10. Take each seedling out of the jar with a sterile pair of tweezers.

***Note:*** Start with at least 100 individual seedlings to ensure positive results.

**CRITICAL:** Work with seedlings in small batches. Removing all seedlings at once causes rapid desiccation, particularly during excision steps.

11. Dissect the seedling into 4 individual parts with a sterile scalpel.

***Note:*** Bigger hypocotyls/cotyledons can be separated into two pieces. Small sections in the adaxial side (perpendicular to the cotyledon axis) can promote callus formation.

**CRITICAL:** Cotyledons are cut once at the tip and once right after the base. Hypocotyls are cut at least 1 cm above the primary root and 1 cm below the shoot apical meristem. The middle part is used, while the root is thrown away. Excisions should be clean. For inexperienced researchers, we recommend discarding the middle hypocotyl section, as regeneration from this region can complicate identification of transgenic shoots.

12. Place the cotyledons and hypocotyls on Preculture – Co-cultivation medium (2ZA) medium for 2 days in a controlled environment plant growth chamber at 23°C–25°C, with a 16/8 light/dark photoperiod, and approximately 100 μmol/m^2^ s full spectrum light.

***Note:*** Place cotyledons with their abaxial side (light green) facing upwards. Cotyledon placement is crucial for efficient infection by *Agrobacterium tumefaciens*.,^5^ likely because the slightly concave surface facilitates bacterial penetration into the tissue. Ensure all explants contact the medium and avoid stacking, which causes desiccation. Dishes can be densely populated at this stage.

### *Agrobacterium tumefaciens* preparation and co-cultivation with the explants

**Timing: 2 days**

In this step, we describe how to grow and precondition *Agrobacterium tumefaciens* carrying a binary plasmid in order to maximize the efficiency of T-DNA transfer into tomato cotyledons and hypocotyls.

13. Start with a fresh plate of *Agrobacterium tumefaciens* transformed with your binary plasmid of choice.

***Note:*** If *de novo* transformation is required, allow 1–2 additional days. Alternatively, use an *Agrobacterium tumefaciens* glycerol stock [20% (v/v) glycerol, -80°C], carrying the binary vector of interest. Fill up a sterile 200 mL Erlenmeyer flask with 50 mL of liquid Yeast Extract Peptone - YEP medium and add the appropriate antibiotics.

14. Inoculate liquid medium containing the appropriate antibiotics with *Agrobacterium tumefaciens* carrying the binary plasmid and incubate for two days at 28°C in a shaking incubator (200 rpm).

***Note:*** GV3101 strain carries the *rpoB* gene which encodes Rifampicin resistance in its C58 chromosomal backbone, along with a helper plasmid pMP90 which encodes Gentamycin resistance through the *aac(3)-Ia* gene. Our binary plasmid of choice carries the *aphA-3* gene which confers resistance to Kanamycin. It is recommended to use all three antibiotics. However, due to the stability of the helper plasmid Gentamycin can be omitted.

15. Transfer the liquid culture in a 50 mL sterile Falcon tube and centrifuge at 1,000 g for 20 min at 23±2°C.
16. Discard the supernatant and resuspend the pellet in 10 mL of freshly prepared liquid Preculture – Co-cultivation medium (2ZA).
17. Measure the OD_600nm_ and dilute your sample with the same media to bring the reading down to 0.2–0.4.

***Note:*** Always use the same buffer as the blank. Approximately 25 mL of *Agrobacterium tumefaciens*-containing culture is required per full Petri plate of tomato cotyledon and hypocotyl pieces. 1–2 new Falcon tubes are needed during this step.

18. Let the bacterial culture acclimate in the new media for 1–2 h in dark room at 23±2°C on a shaker.

***Note:*** Wrap the conical tubes with aluminum foil or use black conical tubes. Each 50-mL tube should contain no more than 25 mL. Loosen the lid slightly and secure it with tape to allow adequate gas exchange.

**CRITICAL:** Do not omit this step as it increases the virulence of the *Agrobacterium tumefaciens*.

19. Move the excised cotyledons and hypocotyls in an empty Petri dish and fill it up with the liquid culture. Wrap Petri dishes tightly with aluminum foil and transfer them on a shaker at 23±2°C with mild rocking (around 100 rpm for 3 mm horizontal orbiter) for about 30 min.
20. Use any kind of sterile gauge to dry the pieces from excess liquid and quickly put them back in the Preculture – Co-cultivation medium (2ZA).

**CRITICAL:** Do not omit this step as decreases the *Agrobacterium tumefaciens* load and limits the frequency of its overgrowth.

21. Wrap the Petri dishes with aluminum foil and incubate them for 2 days at 20°C–22°C.

**CRITICAL:** Do not incubate at temperatures above 25°C as it significantly decreases the T-DNA transferring capacity of *Agrobacterium tumefaciens*.^9^

### Recovery of explants and bacterial overgrowth restriction

**Timing: 2 weeks**

In this step, we describe a simple strategy that allows time for the transformed plant tissue to express the selection resistance gene while actively restricting *Agrobacterium tumefaciens* overgrowth. This is the most common and detrimental problem in tissue culture experiments. From this step onward, we eliminate the need for auxin by introducing the antimicrobial agent Timentin™, a combination of the penicillin antibiotic ticarcillin disodium and the β-lactamase inhibitor clavulanate potassium.

***Note:*** Timentin^TM^ serves a dual role. It is a potent general microbicide without the negative side effects of other commonly used beta-lactam antibiotics, such as callus hyperhydricity induced by Cefotaxime.^10^ Furthermore, ticarcillin's degradation product, thiophene acetic acid, is an auxin analog recently demonstrated to promote organogenesis in tomato.^6^ In combination with *trans*-zeatin, Timentin^TM^ promotes callogenesis and shoot proliferation more rapidly and efficiently.

22. Transfer the explants in freshly prepared solid Regeneration medium (2Z500T) Petri dishes and incubate for a week at controlled environment of a plant growth chamber at 23°C–25°C, with a 16/8 light/dark photoperiod, and approximately 100 μmol/m^2^ s full spectrum light.

***Note:*** Cotyledons are placed with their abaxial side (light green) facing down. Cotyledon placement ensures that the excised tissue surface is directly touching and absorbing the media.

**CRITICAL:** The typical Timentin^TM^ concentration is between 200–300 μg/mL^3^. Here, we introduced a crucial step with high Timentin^TM^ concentration that significantly decreases *Agrobacterium tumefaciens* overgrowth.

23. Transfer the explants in freshly prepared solid selective Regeneration medium (2Z500TK) Petri dishes and incubate in the same conditions for a week.

**CRITICAL:** Add the appropriate antibiotics to the medium as required. In our case, we used Kanamycin at 50 μg/mL (pICH86988 binary plasmid). If numerous untransformed plants escape selection, increase the Kanamycin concentration to 100 μg/mL. We have also successfully used Hygromycin B at 5 μg/mL; however, we highly recommend Kanamycin over Hygromycin B when working with tomato. For the cultivar Moneymaker, we recommend the same 2-fold increase in Kanamycin concentration (to 100 μg/mL).

24. Transfer the explants in freshly prepared solid selective Regeneration medium (2Z250TK) Petri dishes and incubate in the same conditions. Change to fresh Petri dishes once per week.

***Note:*** Visible signs of plant stress including yellowing, browning and dying of the tissue will be observed after extended periods of incubation on 500 μg/mL Timentin^TM^.

**CRITICAL:** Frequent plate-changes in fresh Petri dishes of plant material are essential. Once bacterial overgrowth is visible, discard the dish and begin again with fresh explants from newly co-cultivated material.

### Selection of green calli

**Timing: 1 month to variable**

In this step, we renew the media while waiting for the transformed tissue to regenerate, promoting callus development and shoot growth.

***Note:*** The conditions in the growth chamber are kept stable as mentioned in the previous step.

25. Transfer the explants in freshly prepared solid selective Regeneration medium (2Z250TK) Petri dishes and incubate in the same conditions. Change to fresh Petri dishes every week.
26. After about two weeks, explants will show differential viability: some will become necrotic (brown-black), while others remain green. Select viable green explants and transfer them onto less densely populated plates.

**CRITICAL:** Immediately discard dead tissue as it is a source for *Agrobacterium tumefaciens* overgrowth. Always discard pieces that have visible bacterial overgrowth.

After two weeks, calli of varying morphologies will develop on some explants. Select calli that are green, compact, and show active growth, and transfer them to fresh plates at lower density to allow adequate space for shoot development.

***Note:*** Some calli might become bigger than 2-3 cm in diameter while others might be hard to distinguish with naked eye.

### Regenerated explant excision and growth in non-selective media

**Timing: 2 months to variable**

In this step, we excise well-developed shoots and transfer them to a hormone-free, non-selective medium for root development.

***Note:*** The conditions in the growth chamber are kept stable as mentioned in the previous step.

27. Fill sterile jars with fresh Rooting medium (250TMS/2).
28. Using a sterile scalpel, excise well developed shoots and place them gently inside the medium to grow and eventually root. Transfer shoots to new jars once per 2 weeks.

***Note:*** Start with 5–10 explants per jar, reducing density as shoots develop. Do not discard calli, as they may continue to produce additional shoots. Some browning on the callus surface is normal, as calli are not homogeneously active. Expect to observe a mixture of dead tissue, dead calli, brown-greenish calli, and shoot-producing calli. Group calli by developmental stage and discard only the non-producing tissue and calli.

**CRITICAL:** Make excisions just above where the shoot emerges from the callus. Well-developed shoots are those with at least one node or a visible shoot apical meristem. Do not leave shoots on calli for extended periods, as they will develop stress symptoms and ultimately die. The excised shoot base should be submerged in medium, while the leaves should be above the medium.

29. Once the shoots start rooting, separate them in individual jars. When the root is well developed proceed to the next step.

***Note:*** A well developing root grows actively inside or on top of the nutrient and has a lot of secondary growth.

**CRITICAL:** This is the only step where plants can overcome infections by fungi and bacterial overgrowth. When you see signs of infection, proceed to the next step immediately.

### Plant hardening, potting, and growing

**Timing: 2 months to variable**

In this step, we transfer generated plants from the jars to pots, where they will grow and produce fruits.

***Note:*** Plants experience significant environmental changes when transferred from the plant growth chamber to the walk-in growth chamber or greenhouse. Protect plants from extreme conditions such as direct sunlight for at least two weeks. Maintain temperature at 23 ± 2°C during the day with a slight reduction at night. Relative humidity of 70–80%, well-aerated conditions, and adequate lighting are recommended. Tomato plants are susceptible to many diseases and insect infestations; therefore, precautionary measures such as sticky traps and appropriate pest control applications are essential.

30. Select a well-draining, rich soil mix and gently pot the generated plantlets in plastic propagation containers.

***Note:*** In our experience, the best soil mixes for tomato are pre-fertilized peat-based substrates (black peat) rich in perlite. Avoid substrates rich in coconut coir, as they retain excessive water that can stress or kill young plants.

31. Cover the pots with clear plastic wrap or place them inside a propagator to maintain high humidity for at least 3 days. Over the following days, gradually remove the cover and transfer plants to their final positions.

***Note:*** Some plant loss during hardening is normal. However, if mortality exceeds 20%, reassess potting mix composition and environmental conditions. Take into consideration that transgenic lines may show increased sensitivity to environmental stresses compared to wild-type plants.

**CRITICAL:** This acclimation step is critical for survival. Plants adapted to the high-humidity jar environment will die without gradual transition to ambient conditions. Genotype should inform acclimation protocols, as transgenic or CRISPR knockout lines may be more sensitive to environmental stress. Avoid overwatering; excess moisture causes root rot and mortality.

32. Observe the growth of your tomato plants and adjust your watering schedule based on their stage and environmental conditions. Typically, water 2–3 times per week to maintain moist soil.

***Note:*** During the regeneration process, some regenerated plants exhibit phenotypes that deviate from normal plants, mainly in stem and leaf morphology. Take into consideration that these plants typically revert to normal tomato phenotype within several days after potting.

**CRITICAL:** Discard plants exhibiting multiple main stems, as these are likely chimeric, containing sectors with different genotypes due to independent T-DNA integration events or wild-type cell lineages.

### T-DNA insertion confirmation by PCR

**Timing: 1–2 days**

In this step, we extract genomic DNA from each plant and confirm stable T-DNA integration.

***Note:*** DNA extraction from tomato tissue and Polymerase Chain Reaction (PCR) confirmation of generated lines is not the main aspect of this protocol; thus, these procedures will be briefly discussed to provide researchers with a complete guide for the entire transformation process.

33. In about 6 months, you expect more than 20–50 potted, well developed individual plants. Use a 1.5 mL Eppendorf tube to obtain a leaf disc and extract the DNA using the following Edward’s-isopropanol method^11^:
  ***Note:*** Plants generated by the tissue culture method are generally considered T_0_ plants because they originate from somatic cells of the mother plant that have been cultured and regenerated *in vitro*.
  a. Prepare 50 mL of Edward’s DNA extraction buffer (final concentration 200mM Tris-HCl pH=7.5, 250mM NaCl, 25mM EDTA, 0.5% SDS).
  b. Add 600 μL of DNA extraction buffer and grind the tissue with a plastic micro-pestle.
  c. Centrifuge at 11,000g for 10 min at 23±2°C.
  d. Transfer 400 μL of the greenish supernatant to a new 1.5 mL Eppendorf tube and discard the pellet.
  e. Fill up to 1.5 mL with ice-cold isopropanol and gently invert the tubes.
  f. Centrifuge at 11,000g for 15 min at 4°C.
  g. Discard the supernatant and fill up to 1.5 mL with ice-cold 70% (v/v) ethanol.
  h. Centrifuge at 11,000g for 10 min at 4°C.
  i. Discard the supernatant. Spin down if necessary and discard the supernatant again.
  j. Let the white pellet dry out for 10–20 min.
  k. Fill with 200 μL sterile dH_2_O and mix by pipetting 2–3 times. Do not completely dissolve the pellet. Store at −20°C.
34. Design and obtain the appropriate primer pair for your PCR.

***Note:*** Design primer pairs where at least one primer is specific to the T-DNA construct and does not anneal to the tomato genome (verify using NCBI Primer-BLAST). Target non-plant sequences such as viral promoters or selection marker cassettes. Maintain amplicon size < 1 kb for optimal PCR efficiency.

35. Assemble and run your PCR reaction using < 2 μL of DNA template.

***Note:*** If PCR reaction fails to produce the expected band, reduce the volume of template used. Leaf metabolites often inhibit polymerase enzymes.

**CRITICAL:** Select a DNA polymerase that is resistant to PCR metabolic inhibitors like Thermo Scientific Phire, DreamTaq, or similar.

36. Analyze your generated T_0_ lines by Agarose gel electrophoresis.

***Note:*** Ensure that you always use a positive control (plasmid) and negative control (wild-type plant). Due to residual plant metabolites or template impurities, PCR product migration may not precisely match expected sizes based on the DNA size marker. Aim to confirm at least 10–20 independent transgenic lines.

**Figure 1:**
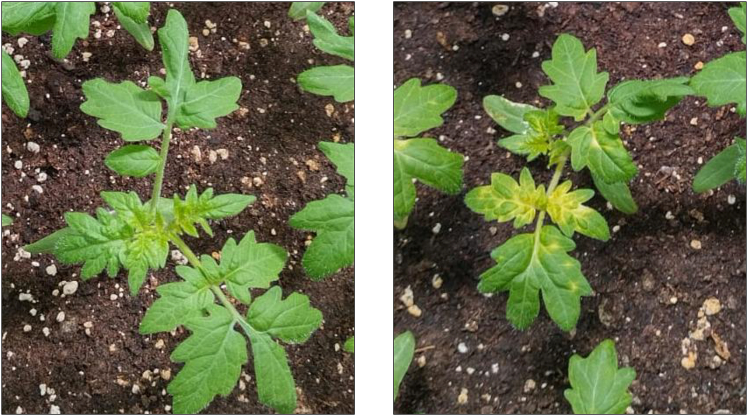
Phenotypic selection of transgenic tomato seedlings via Kanamycin foliar application Representative photographs of tomato seedlings one week following three consecutive Kanamycin foliar spray applications (see Materials and Methods for concentration and spray schedule). Left panel: Kanamycin-resistant transgenic plant (T-DNA positive) showing normal green foliage and healthy growth with no visible phytotoxic effects. Right panel: Non-transgenic (wild-type) control plant displaying characteristic chlorosis and leaf yellowing due to Kanamycin-induced inhibition of chloroplast development. This differential response enables rapid visual screening and early identification of putative transgenic lines prior to molecular confirmation.

37. To complete your experiment, obtain seeds from T_0_ positive lines and test again in T_1_ generation by PCR and Agarose gel electrophoresis or the optional foliar spray assay desdcribed below.
  ***Optional:*** For Kanamycin resistant plants, an additional confirmation strategy can be used:
  a. Prepare a solution containing 0.5 mg/ml Kanamycin and 0.05% (v/v) non-ionic surfactant (e.g., Sapogenat).
  b. Spray young plants (3–5 weeks after germination) until their foliage is completely covered for 3 consecutive days.
  c. Evaluate the plant response after a couple of days.
    ***Note:*** T-DNA free plants exhibit bleaching symptoms, whereas Kanamycin-resistant plants remain green and symptom-free. An example of expected symptoms can be seen in Figure 1.
    **CRITICAL:** We have noticed that some of our T_0_-positive lines are chimeric, which is problematic for many applications, including CRISPR knockouts. We recommend advancing to T_1_ generation to obtain non-chimeric lines. Do not conduct phenotyping experiments on T_0_-positive lines, as we have observed stress-related phenotypes that are irrelevant to the transgene or mutation. We do not record the callus origin of individual shoots or resulting T_0_-positive lines because many calli are chimeric. Multiple shoots arising from the same callus may harbor different T-DNA integration events. Thus, no definite determination can be made about the number of individual T-DNA insertion events without further experimental work (e.g., whole-genome sequencing).

## Expected outcomes

This protocol was developed to obtain *Agrobacterium tumefaciens-*mediated stable transformation lines of tomato plants in under six months. The expected outcome is at least 10–20 generated PCR-positive lines from each complete experiment. We highlight that using this protocol, one researcher has produced more than 10 independent tomato lines, such as proteins fused to fluorophores, CRISPR knockouts, and Moneymaker mutant lines, over the past 2 years.

Since this protocol is not associated with a primary research manuscript, we created some overexpressing Enhanced Yellow Fluorescent Protein (EYFP) MicroTom tomato transgenic lines as a proof of concept. We amplified the EYFP DNA from pICSL50005 (Addgene #117536) using the primers “taggtctctaatggtgagcaagggcgag” and “gtggtctcaaagctcacttg” and the Q5®High-Fidelity DNA polymerase (New England Biolabs – suggested protocol). Then, we ran gel electrophoresis and extracted the PCR fragment using the Nucleospin® Gel and PCR Clean-up kit (Macherey-Nagel – suggested protocol). The final vector was assembled through the GoldenGate method using the BsaI -HF®v2 restriction enzyme (New England Biolabs – suggested protocol). The empty binary vector used for this purpose was pICH86988 (Addgene #48076).^12^^,^^13^ The T-DNA region expresses *neomycin phosphotransferase II* gene (NPTII) under the control of the nopaline synthase (NOS) promoter, which confers resistance to Kanamycin. In addition, the inserted EYFP gene is overexpressed under the control of the CamV35S promoter and the Ω leader. The final construct was confirmed through Sanger sequencing and transformed in GV3101 *Agrobacterium tumefaciens*.

**Figure 2:**
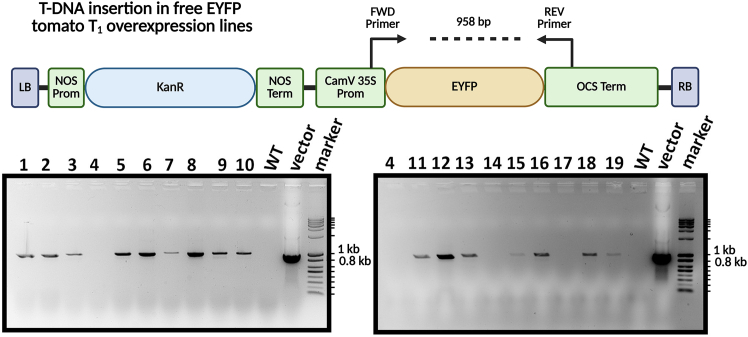
T-DNA construct design and molecular confirmation of transgenic tomato lines Upper panel: Schematic representation of the binary T-DNA vector used for tomato transformation. The construct contains a Kanamycin resistance cassette (KanR) driven by the nopaline synthase promoter (NOS Prom) and terminator (NOS Term) for plant selection, and an Enhanced Yellow Fluorescent Protein (EYFP) reporter gene under the control of the cauliflower mosaic virus 35S promoter (CaMV 35S Prom) and octopine synthase terminator (OCS Term). LB and RB denote left and right T-DNA borders, respectively. Forward (FWD) and reverse (REV) primer binding sites are indicated by arrows, amplifying a 958 bp product spanning the *EYFP* gene region. Lower panel: PCR-based genotyping of putative T_0_ transgenic lines. Genomic DNA from independent transgenic candidates (lanes 1-10 and 11-19) was amplified using the primers shown in the upper panel. Wild-type (WT) serves as a negative control showing no amplification, while the binary vector DNA serves as a positive control. DNA size markers are shown in the rightmost lanes of each gel. Amplification of the expected ∼958 bp band confirms successful T-DNA integration in positive transgenic lines (visible in lanes 5–7, 9–10 on the left gel and lanes 11, 15–16, 19 on the right gel).

In Figure 2, we present PCR and agarose gel electrophoresis confirmation of the first 18 individuals tested, along with the design of the primer pair on the inserted T-DNA fragment. We achieved up to approximately 90% efficiency in generating T_0_ lines with this construct. However, efficiency varies considerably between vectors, experimental replicates, and researchers. In our experience, pCAMBIA backbone vectors with the Kanamycin resistance gene yield as low as 38% PCR-positive lines, while those with the Hygromycin B resistance produce below 10% PCR-positive lines.

**Figure 3:**
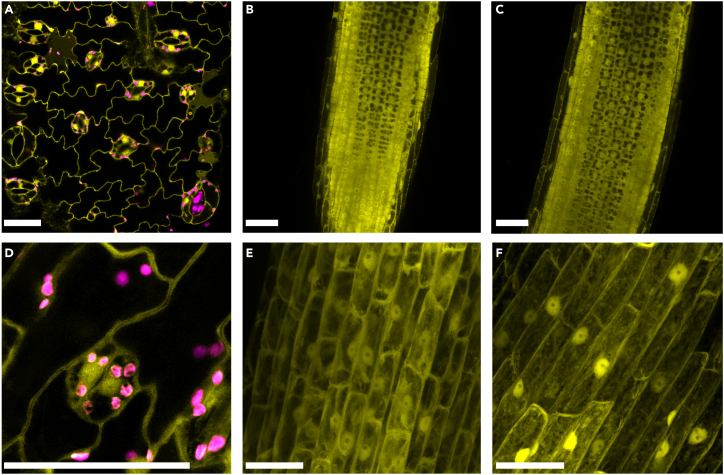
Confocal imaging of EYFP fluorescence in different tomato tissues Confocal micrographs showing EYFP fluorescence (yellow) in various tomato tissues (from several individual lines). (A) Cotyledon epidermal cells. (B and C) Longitudinal sections of primary roots showing EYFP expression across developmental zones. (D) Leaf epidermal cells showing EYFP signal and endogenous autofluorescence (magenta) from chloroplasts. (E and F) Maximum intensity z-projections of root cortical cells displaying subcellular EYFP localization patterns. Images were acquired using a Leica SP8 confocal laser scanning microscope equipped with a HC PL APO 40×/1.30 Oil CS2 - NA 1.30. EYFP was excited at 514 nm (sometimes with 488 nm) and detected between 525–560 nm; endogenous chlorophyll autofluorescence was collected between 650–700 nm using the same channel through Hybrid Detectors (HyD). Z-stacks were acquired with 0.5 μm step size (>10 steps) and processed using Leica LAS X software. Representative micrographs are shown from N > 3 independent experiments from several individual lines. Scale bars: 50 μm.

**Figure 4:**
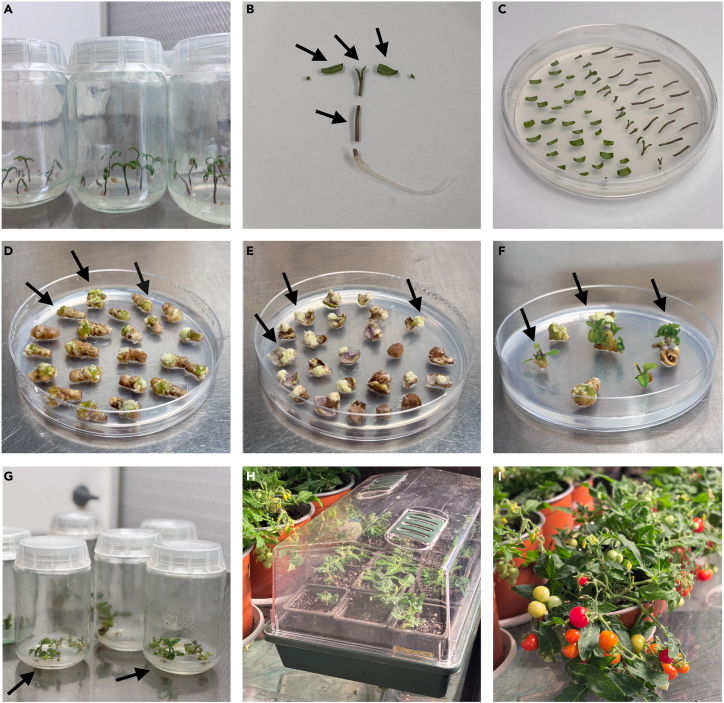
Visual overview of workflow stages (A) Five-day-old seedlings displaying fully expanded cotyledons prior to the emergence of first true leaves. (B) Representative seedling sections; arrows indicate specific tissue regions utilized for explant preparation. (C) Excised plant tissues cultured on 2ZA medium with cotyledon abaxial surfaces oriented upward. (D) Hypocotyl segments cultured on 2Z250TK medium exhibiting callus formation, indicated by arrows highlighting light green calli. (E) Cotyledon explants grown on 2Z250TK medium (cotyledons flipped for imaging); arrows denote sites of callus induction (light green). (F) Shoot organogenesis observed from hypocotyl- and cotyledon-derived calli on 2Z250TK medium; arrows mark shoots ready for excision. (G) Explants transferred to 250TMS/2 rooting medium; arrows point to emergent roots. (H) Plantlets undergoing acclimation in a controlled propagator environment. (I) The PCR-confirmed transgenic lines (from Figure 2) transplanted into 2-liter pots showing mature fruit development.

To confirm T-DNA integration stability, we picked six random T_0_-positive individuals (1, 3, 7, 10, 14, 16) and propagated them. After 2 months, we collected the fruits, sterilized the seeds, and grew them on solid MS/2 media. After 10 days, we visualized EYFP fluorescence using a 40 x oil immersion objective (NA = 1.30) on a Leica SP8 confocal microscope excited with a 514 nm argon laser (sometimes with 488 nm). EYFP emission was detected at 525-560  nm, while chlorophyll autofluorescence was detected at 650-700 nm using Hybrid Detectors (HyD). Figure 3 shows images of both root and leaf tissue are presented confirming the stability and expression of the inserted T-DNA. Figure 4 shows representative images from each major step of the workflow, providing visual references for researchers.

## Limitations

This protocol was optimized for tomato cultivar MicroTom using Kanamycin as a selection marker under the control of NOS promoter. This protocol works for other tomato cultivars, such as Moneymaker, either as is or with slight adjustments, mainly to the antibiotic concentrations, as suggested above. We chose MicroTom and Moneymaker deliberately because they represent two contrasting and widely used tomato cultivars: MicroTom as a model laboratory variety with compact growth and rapid life cycle, and Moneymaker as a standard commercial cultivar with typical indeterminate growth. Together, these cultivars capture the major physiological and developmental differences that could influence our study, providing a robust test of the protocol. We cannot exclude the possibility that other varieties may require further optimization for successful regeneration.

Kanamycin remains the gold standard for tomato tissue culture, although when multiple rounds of transformation events are essential, other options should be explored. We have successfully used the same protocol for Hygromycin B selection marker under the control of the CaMV 35S promoter although, shoot and calli development were much slower. Simultaneously, explants showed significant signs of discoloration and stress, leading to much lower efficiency of positive line generation. In our experiment, Hygromycin B selection gives a fivefold decrease in PCR-positive lines, while the process necessitates more meticulous tissue handling by the researcher. We have not done yet trials with Phosphinothricin or Glufosinate herbicides as selection markers.

We emphasize that the efficiency varies significantly depending on the T-DNA construct, and researcher experience. Moreover, unforeseen factors such as crucial step delays, fungal infections, or unexpected bacterial overgrowth, etc., can ruin months of work. Therefore, we suggest conducting multiple independent transformation experiments before in-house optimization of the protocol.

The use of Timentin^TM^ is crucial for the success of our protocol. Timentin^TM^ is a mixture of ticarcillin, a β-lactam antibiotic that inhibits bacterial cell-wall synthesis, and clavulanic acid, a β-lactamase inhibitor that protects ticarcillin from bacterial enzymes. However, in cases of high costs or limited accessibility, we recommend Cefotaxime, although more frequent media replacement may be required due to reduced stability. Another common alternative is Carbenicillin, but we advise against its use, as most common *Agrobacterium tumefaciens* strains such as GV3101, AGL-1 and C58C1 are naturally resistant to this antibiotic.

We do not include statistical analysis of regeneration efficiency in this protocol, as it falls outside our scope and is highly variable, as explained above. Our focus here is on identifying true-positive lines. In addition, we do not provide information about ploidy validation which is sometimes necessary for the discovery of new phenotypes in tomato after tissue culture. For additional information on ploidy determination, we suggest Wang et al. 2025 or similar protocols.^4^

## Troubleshooting

### Problem 1

*Agrobacterium tumefacients* overgrowth on plant tissue, causing tissue damage or death.

### Potential solution

• Completely dry the excised tissue on sterile gauge after submerging to *Agrobacterium tumefaciens* solution in *step* 14. Repeat again before *step* 16 if necessary.
• Dip the excised parts in 1 mg/mL Timentin^TM^ solution and immediately wash with sterile dH_2_O before *step* 16.
• If a callus shows *Agrobacterium tumefaciens* (over)growth, immediately remove and discard. Place the rest of the plant tissue in new media, mark the plate and do not mix it with other “clean” ones.
• Incubate at 32°C for a week at *step* 16 to discourage *Agrobacterium tumefaciens* growth.
• Do not omit frequent media changes on both Petri dishes and jars as stated above.
• Remove obviously necrotic tissue from viable explants. Quarantine uncertain explants in a separate dish and reassess after several weeks.
• Avoid overfilling Petri dishes. Use 15–20 mL of medium per dish to ensure adequate aeration and minimize humidity-related fungal infection.
• Maintain uniform air circulation in the growth chamber to avoid localized hot spots (e.g., directly beneath lamps), which increase humidity and promote bacterial contamination.
• Lower the growth chamber temperature to 20–22°C and reduce light intensity. These adjustments decrease photosynthetic and respiration rates as well as medium evaporation, thereby reducing humidity levels.
• Verify Timentin™ activity. A fresh stock solution is light yellow, but turns dark yellow/brown upon degradation. If discolored, prepare a fresh stock solution.

### Problem 2

Low regeneration frequency, tissue necrosis, no callus or shoot formation.

### Potential solution

• Verify *trans*-zeatin activity (prepare fresh stock).
• Optimize seedling stage before starting your experiment before *step* 5.
• Verify *Agrobacterium tumefaciens* initial OD_600nm_ at *step* 11.
• Check all the pH of the media and do not omit sucrose use when necessary.
• Check antibiotic concentrations and optimize accordingly. With Hygromycin B, both callus size and subsequent shoot size are typically smaller.

### Problem 3

Poor rooting, explants die after excision.

### Potential solution

• Ensure that the blade or tissue manipulation does not destroy the explant. A common mistake is thermal damage from burning tissue with blades or tweezers, especially at this stage. Cool tools to room temperature by dipping them in medium for at least 10 s before each use (step 29).
• Ensure you excise/transfer shoots with visible shoot apical meristems at *step* 23. Isolated leaves lacking meristems rarely develop into plants.
• Ensure you do not use antibiotics, especially Kanamycin, during rooting at *step* 22.

### Problem 4

Plant death after soil transfer.

### Potential solution

• Do not omit hardening suggestions at *step* 26.
• Ensure adequate drainage by selecting appropriate substrate or increasing perlite concentration (*step* 25).
• Gently cover the roots without covering the above ground part with soil at *step* 25.
• Do not water excessively during the first weeks at *step* 27.

### Problem 5

Somaclonal variation or unwanted mutations may be introduced during the tissue culture and regeneration process.

### Potential solution

• Do not extend the tissue culture time more than necessary.
• Generate at least 10–20 T_0_ positive lines and proceed to next generation before conducting your experiments.
• Discard plants exhibiting aberrant phenotypes, such as shoot-like structures without meristems or “leafy regenerants”, unless the same phenotype is observed in multiple independent positive lines.

## Resource availability

### Lead contact

Further information and requests for resources and reagents should be directed to and will be fulfilled by the lead contact, Panagiotis Nikolaou Moschou (panagiotis.moschou@uoc.gr).

### Technical contact

Technical questions on executing this protocol should be directed to and will be answered by the technical contact, Fanourios Mountourakis (bio.mountourakis@gmail.com).

### Materials availability

This study generated tomato (cv MicroTom) T-DNA lines overexpressing enhanced yellow fluorescent protein. Seeds and the T-DNA plasmid are readily available upon request to Panagiotis Nikolaou Moschou (panagiotis.moschou@uoc.gr).

### Data and code availability

This study did not generate new data and code.

## Acknowledgments

This work was supported by grants from Horizon Europe European Research Council (ERC 101126019 to P.N.M.) and European Union and Greek national funds through the Operational Program Competitiveness, Entrepreneurship, and Innovation (T2ΕΔΚ–00597 under the call RESEARCH–CREATE–INNOVATE “BIOME” to P.N.M.). Views and opinions expressed are those of the authors only and do not necessarily reflect those of the European Union or the European Research Council Executive Agency. Neither the European Union nor the granting authority can be held responsible for them. The graphical abstract and figures for this publication were created with BioRender (Mountourakis, F. 2025).

## Author contributions

F.M. conceived and carried out the experiments. F.M. wrote the manuscript in consultation with S.F. and P.N.M. P.N.M. supervised the project.

## Declaration of interests

The authors declare no competing interests.
